# Vaccination Prioritization Strategies for COVID-19 in Korea: A Mathematical Modeling Approach

**DOI:** 10.3390/ijerph18084240

**Published:** 2021-04-16

**Authors:** Yongin Choi, James Slghee Kim, Jung Eun Kim, Heejin Choi, Chang Hyeong Lee

**Affiliations:** Department of Mathematical Sciences, Ulsan National Institute of Science and Technology, Ulsan 44919, Korea; yongin9@unist.ac.kr (Y.C.); jameskim@unist.ac.kr (J.S.K.); jkimmath1@unist.ac.kr (J.E.K.); chlgmlwls@unist.ac.kr (H.C.)

**Keywords:** COVID-19, vaccination priority strategy, mathematical modeling, social distancing

## Abstract

Coronavirus disease 2019 (COVID-19) vaccination has recently started worldwide. As the vaccine supply will be limited for a considerable period of time in many countries, it is important to devise the effective vaccination strategies that reduce the number of deaths and incidence of infection. One of the characteristics of COVID-19 is that the symptom, severity, and mortality of the disease differ by age. Thus, when the vaccination supply is limited, age-dependent vaccination priority strategy should be implemented to minimize the incidences and mortalities. In this study, we developed an age-structured model for describing the transmission dynamics of COVID-19, including vaccination. Using the model and actual epidemiological data in Korea, we estimated the infection probability for each age group under different levels of social distancing implemented in Korea and investigated the effective age-dependent vaccination strategies to reduce the confirmed cases and fatalities of COVID-19. We found that, in a lower level of social distancing, vaccination priority for the age groups with the highest transmission rates will reduce the incidence mostly, but, in higher levels of social distancing, prioritizing vaccination for the elderly age group reduces the infection incidences more effectively. To reduce mortalities, vaccination priority for the elderly age group is the best strategy in all scenarios of levels of social distancing. Furthermore, we investigated the effect of vaccine supply and efficacy on the reduction in incidence and mortality.

## 1. Introduction

Since the first case reported in Wuhan, China, in December 2019, Coronavirus Disease 2019 (COVID-19) has rapidly spread worldwide. COVID-19 is caused by severe acute respiratory syndrome coronavirus 2 (SARS-CoV-2). The cases of the disease display similar symptoms to those of Middle East Respiratory Syndrome and Severe Acute Respiratory Syndrome, such as fever, dry cough, dyspnea, and diarrhea [1]. The disease has been characterized as a pandemic by the World Health Organization (WHO) on 11 March 2020 [2]. On 17 February 2021, it was reported that more than 108.8 million people were infected with COVID-19, and more than 2.4 million casualties were recorded across 223 countries [3]. To reduce the spread of the disease while COVID-19 vaccines were not available, countries initially used contact tracing [4] and later implemented non-pharmaceutical interventions (NPIs), which include social distancing (SD), wearing masks, epidemiological surveys, and work/school closure, following the guidelines made by their own government and by WHO.

On 16 February 2021, there have been 84,325 cases with 1534 casualties in Korea; 57.1% of these cases were from the densely populated metropolitan area—26,484 cases from Seoul and 21,648 cases from Gyeonggi Province [5]. As contact tracing was not sufficient to prevent the spread of the disease, NPIs have been implemented differently across regions in Korea, and owing to high floating population and population density, reinforced measures for infection control have been implemented in Seoul and Gyeonggi Province. Many social events and gatherings were limited or prohibited, work-at-home was enforced or advised, and schools were closed frequently. However, despite these efforts, which are not without economic, social, and psychological sacrifices caused by NPIs and ethical issues that accompany contact tracing [6], COVID-19 cases still exist, and ending this dynamic does not seem probable with the current level of efforts. It is expected that effective vaccination can improve this situation.

As of 17 February 2021, four different COVID-19 vaccines have been approved for full use, and six different vaccines have already been approved for early or limited use [7]. The Korean government has released the plan for vaccination schedule, in which the administration starts in February until December 2021 [8]. A description of the vaccines is given in Table 1. The government plans to finish the administration of the first dose for the public by the third quarter of 2021 and accomplish herd immunity in November 2021. Currently, Korea has secured the access of COVID-19 vaccines from five different pharmaceutical companies—Pfizer, AstraZeneca, Moderna, Johnson & Johnson, and Novavax—for 79 million people [8,9], which is approximately 152.5% of the total population.

Recent studies have reported that the susceptibility [15,16], infectivity [16], severity [17], and fatality [18] of COVID-19 vary with age. Thus, the number of incidences, severe patients, and deaths will vary depending on how the vaccination priority is determined for each age group. Besides essential workers and patients in critical condition, Centers for Disease Control and Prevention recommends vaccination priority for the elderly people [19].

The Korean government plans to prioritize vaccination by dividing the entire population, excluding frontline essential workers and patients in critical condition, into different age groups [8]. To reflect this plan and simulate the effect of vaccination under various scenarios, we construct a mathematical model for describing the transmission of COVID-19 with the vaccination prioritization of four age groups: 0–19, 20–49, 50–64, and 65 or older. Several studies on mathematical modeling for COVID-19 vaccination have been conducted recently. In References [20,21], the critical vaccination coverage for various hypothetical vaccine efficacy scenarios is estimated in South Africa and Australia, respectively, to control the disease. In References [22,23,24,25], the effect of vaccination in terms of efficacy and coverage is investigated combined with other interventions, such as the rollout speed of COVID-19 vaccine, face mask usage, or SD. Age-structured models are considered in References [26,27,28,29], and optimal vaccine allocation strategies on age groups are identified under various control scenarios. A multi-region epidemic model is constructed in Reference [30], and the optimal control theory is applied to reduce the number of infectious individuals in the targeted domain with an optimal cost. The appropriate price for COVID 19 vaccine is suggested in Reference [31] using a mathematical model.

In this study, we aimed to investigate the effect of vaccination priority strategies for different age groups on infection incidence and mortality under different SD levels implemented in Korea. For this purpose, we first developed an age-structured mathematical model to describe the transmission dynamics of COVID-19 combined with vaccination. Using the age-structured mathematical model, we also compared the effect of age-dependent vaccination priority strategies on various levels of vaccine efficacy and supply.

## 2. Materials and Methods

### 2.1. Epidemiological Data

**Figure 1:**
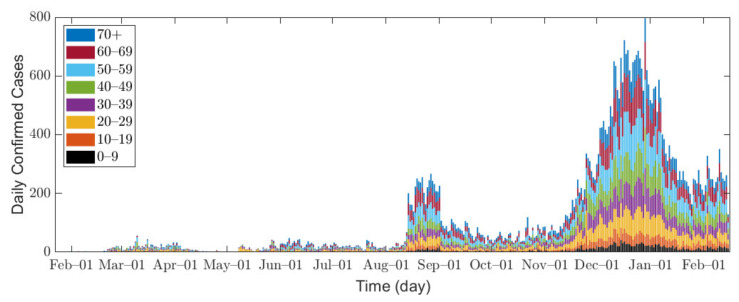
Confirmed cases in Seoul/Gyeonggi area.

In this research, we used data of confirmed COVID-19 cases in Seoul and Gyeonggi Province between 1 February 2020 and 14 February 2021 [32,33]. Because the data for Seoul are provided in age groups of 10 years, we combine the data from both Seoul and Gyeonggi Province into 10-year age groups for consistent interpretation, namely 0–9, 10–19, 20–29, 30–39, 40–49, 50–59, 60–69, and 70 or older (70+). Figure 1 shows the epidemic curve of confirmed COVID-19 cases, and Table 2 shows the total number of confirmed cases for each age group in Seoul/Gyeonggi area.

In Seoul/Gyeonggi area, the number of confirmed cases per day remained below 100 before August 2020 but increased to approximately 200 after mid-August. Since mid-November, a large number of confirmed cases has emerged as winter approaches [34], which can be explained by the fact that the contact time in a confined space has increased, and indoor environments provide a stable condition for SARS-CoV-2 with less sunlight [35].

The infection incidences in Seoul/Gyeonggi area were heavily affected by different levels of SD implemented by the Korean government [36,37,38,39]. The definition of SD levels announced by the government varied in the course of our study. Initially, there were three levels of governmental SD, but, on 7 November 2020, the Korean government revised the governmental SD to five levels as shown in Table S1 in Supplementary Section A1 [40]. In addition, the government’s decision and policy-making does not strictly follow the definitions, as the SD announced by the government is considered as a guideline. For example, elevation to governmental SD level 2 requires a monitoring duration of confirmed cases for at least 1 week at governmental SD level 1.5 [40], but governmental SD level 1.5 only lasted for 5 days [41]. Moreover, governmental SD level 2.5 was reinforced with the prohibition of gathering of five or more people on 24 December 2020 [42], despite the fact that governmental SD level 3 only prohibits the gathering of 10 or more people. For the above reasons, the definite classification of SD was difficult, so referring to the government policies that actually took effect, we set our own criteria for SD as presented in Table 3. In this research, the contact matrix, which characterizes the contact degree between age groups, is the linear combination of the location-specific matrices of workplace, school, household, and other locations [43], and SD levels directly impact the contact matrix by location and age as shown in Table 3; the details of the contact matrix will be explained in Section 2.2 and Supplementary Section B1.

### 2.2. Mathematical Model

**Figure 2:**
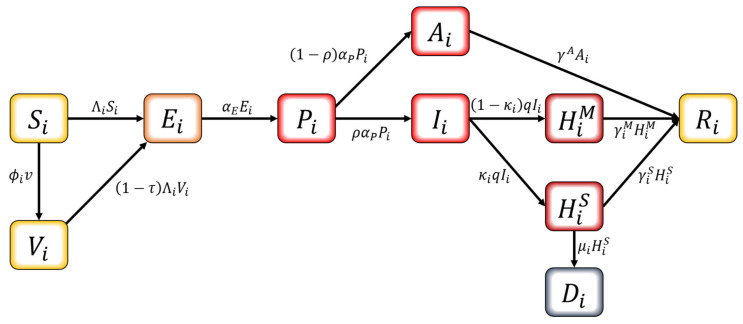
Schematic diagram of the mathematical model.

We developed an age-structured mathematical model to describe the transmission dynamics of COVID-19 with vaccination. In this model, the population is separated into compartments based on their characteristics for each age group i: $S_{i}=\mathrm{susceptible}$, $V_{i}=\mathrm{vaccinated}$, $E_{i}=\mathrm{exposed}$, $P_{i}=\mathrm{pre}-\mathrm{symptomatically}\;\mathrm{infectious}$, $A_{i}=\mathrm{asymptomatically}\;\mathrm{infectious}$, $I_{i}=\mathrm{asymptomatically}\;\mathrm{infectious}$, $H_{i}^{M}=$ hospitalized with mild symptoms, $H_{i}^{S}=$ hospitalized with severe symptoms, $R_{i}=\mathrm{recovered}$ and $D_{i}=\mathrm{dead}$. The age classes i=1,2,…,8 represent individuals aged 0–9, 10–19, 20–29, 30–39, 40–49, 50–59, 60–69, and 70+, respectively. The schematic diagram of the model is shown in Figure 2. The susceptible population can be vaccinated or become infected. Infection can occur for both the susceptible and the vaccinated if pre-symptomatically, asymptomatically, or symptomatically infectious population makes contact. Once considered infected, this population is exposed and becomes pre-symptomatically infectious after the latent period. The pre-symptomatically infectious population does not show any symptom and moves on to either asymptomatically infectious or symptomatically infectious. The asymptomatically infectious population continues to show no symptoms; thus, no isolation is made until they become recovered. On the other hand, the symptomatically infectious population will be hospitalized based on the severity of their symptoms—either mild or severe—once they are confirmed with COVID-19. The hospitalized population is completely prohibited from making contacts with others. Both hospitalized groups recover, except for those who die from a group with severe symptoms.

The differential equations for describing the model are as follows.

(1) $$\begin{array}{l} \dot{S_{i}}=-\Lambda_{i}S_{i}-\phi_{i}v\\ \dot{V_{i}}=\phi_{i}v-\left(1-\tau \right)\Lambda_{i}V_{i}\\ \dot{E_{i}}=\left(S_{i}+\left(1-\tau \right)V_{i}\right)\Lambda_{i}-\alpha_{E}E_{i}\\ \dot{P_{i}}=\alpha_{E}E_{i}-\alpha_{P}P_{i}\\ \dot{A_{i}}=\left(1-\rho \right)\alpha_{P}P_{i}-\gamma^{A}A_{i}\;\\ \dot{I_{i}}=\rho \alpha_{P}P_{i}-qI_{i}\\ {\dot{H}}_{i}^{M}=\left(1-\kappa_{i}\right)qI_{i}-\gamma_{i}^{M}H_{i}^{M}\\ {\dot{H}}_{i}^{S}=\kappa_{i}qI_{i}-\gamma_{i}^{S}H_{i}^{S}-\mu_{i}H_{i}^{S}\\ \dot{R_{i}}=\gamma^{A}A_{i}+\gamma_{i}^{M}H_{i}^{M}+\gamma_{i}^{S}H_{i}^{S}\\ \dot{D_{i}}=\mu_{i}H_{i}^{S}, \end{array}$$

where the infection force $\Lambda_{i}$ for each age group i=1,2,…,8 is $\Lambda_{i}=b_{i}\overset{8}{\underset{j}{\sum }}\left[\frac{m_{ij}\left(\theta_{P}P_{j}+\theta_{A}A_{j}+\theta_{I}I_{j}\right)}{N_{j}}\right]$. The model parameters in Equation (1) are described in Table 4, and $N_{j}$ is the total contact possible population of age group j, equivalently $N_{j}=S_{j}+E_{j}+P_{j}+A_{j}+I_{j}+R_{j}$. Note that $m_{ij}$ is an entry of the contact matrix,$C_{M},$ which is an 8×8 matrix estimated from [36,43] reflecting the actual transmission rates between individuals of different ages in the target area. The detailed computation for contact matrix is given in Supplementary Section B1.

In this study, we find the effective reproduction number $R_{t}$ which measures the mean number of secondary cases infected by an infectious individual. Mathematically, it is computed as $R_{t}=\rho \left(G\right),$ where ρ is the spectral radius of the next generation matrix  G. For model (1), $R_{t}$ is derived as follows:

$$R_{t}=\rho \left(G\right)=\left(\frac{\theta_{P}}{\alpha_{P}}+\frac{\left(1-\rho \right)\theta_{A}}{\gamma^{A}}+\frac{\rho \theta_{I}}{q}\right)\rho \left(M_{A}\right)$$

Here, $M_{A}$ is the matrix computed as follows:

$$M_{A}=diag{\left\{b_{1}\left(S_{1}+\left(1-\tau \right)V_{1}\right),b_{2}\left(S_{2}+\left(1-\tau \right)V_{2}\right),\ldots ,b_{8}\left(S_{8}+\left(1-\tau \right)V_{8}\right)\right\}}_{8}*C_{M}*diag{\left\{\frac{1}{N_{1}},\frac{1}{N_{2}},\ldots ,\frac{1}{N_{8}}\right\}}_{8},$$

where $S_{i}$ is the susceptible population of age group i, $V_{i}$ is the vaccinated population of age group i,$\;C_{M}$ is the 8×8 contact matrix, and $diag{\left\{\;\right\}}_{n}$ denotes the diagonal matrix with n diagonal entries. The detailed derivation of the above formula for $R_{t}$ is given in Supplementary Section B2.

### 2.3. Vaccination Strategies

**Figure 3:**
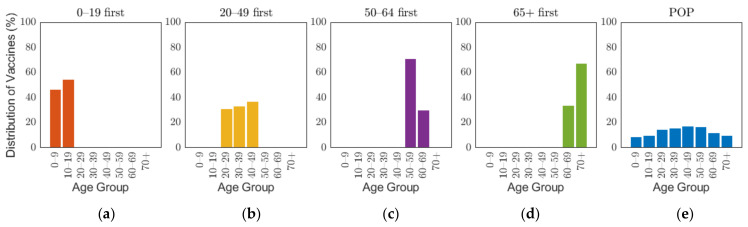
Distribution of vaccine scenarios for five prioritization strategies: (**a**) 0–19 first, (**b**) 20–49 first, (**c**) 50–64 first, (**d**) over 65 first, and (**e**) proportion of population (POP).

The Korean governmental vaccination plan on 28 January 2021 states that [8], excluding frontline essential workers and patients in critical condition, vaccines will be administered based on age groups, such as 18–49, 50–64, and 65 or older, starting with the elderly, and 70% of the population will be vaccinated in 6 months from its start date [45]. Though the vaccination of people under the age of 18 has not been decided, the government is planning to administer the vaccine depending on the clinical outcomes [8]. To emphasize the effects of vaccine administration scenarios based on age groups, and because of non-exact vaccination administration schedule based on the producers, the overall vaccination efficacy was used in this research, which was calculated as 88% by taking the average of efficacy of different vaccines in Table 1 with doses as weights. The details of the calculation are explained in Supplementary A2. As the demographic in our study considered 10-year age groups, we investigated four different vaccination priority strategies, which initially distributed vaccination to target age groups of 0–19, 20–49, 50–64, and 65 or older (65+) by 70% of the target population, and then vaccination was administered to other age groups proportional to their population until 70% of the total population was vaccinated. We will refer this vaccination priority strategies for different age groups 0–19, 20–49, 50–64, and 65+ by “0–19 first”, “20–49 first”, “50–65 first”, and “65+ first”, respectively. Note that our simulation was based on 10-year age groups, but we implemented two vaccination strategies “50–64 first” and “65+ first”, which are not separable by 10-year intervals. To resolve the discrepancy in the age intervals, vaccines distributed to age groups 60–64 and 65–69 were based on population ratio [48]. Four of the five vaccines planned to be introduced in Korea require two vaccine doses per person as shown in Table 1. In this study, we considered the date of the last vaccination as the vaccination date. To compare the case with no prioritization, an additional strategy was considered where vaccination was administered to 70% of all age groups with proportion of population from the start, which will be referred to herein as the POP strategy. Figure 3 illustrates the vaccination allocation for age groups in the five vaccination strategies. The slight difference of distribution of vaccines between age groups within each strategy is caused by the population difference of age groups. The general population ratio between age groups can be observed in the distribution of vaccines in the POP strategy where all age groups were administered with equal coverage rate.

Since the SD can significantly affect the transmission dynamics of COVID-19 [36] and it is very likely that vaccination will be administered while appropriate SD policies are implemented in Korea, we investigated the effect of the five vaccination priority strategies on the number of confirmed cases and mortalities under the assumption of the different SD levels. Furthermore, as the Korean government has criteria for changing the intensity of SD according to the number of confirmed cases [33], we reflected these criteria in the simulation to investigate the effect of the age-dependent vaccination priority strategies on the number of confirmed cases and mortalities.

### 2.4. Parameter Estimation

**Figure 4:**
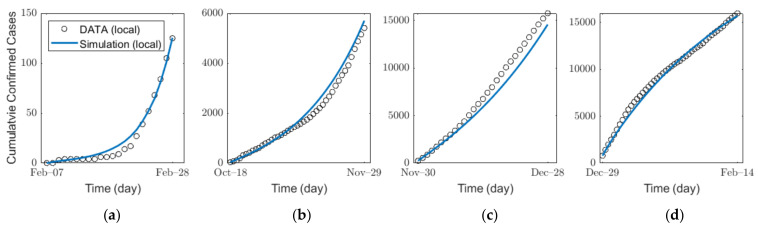
Estimation of the infection probability for different levels of SD: SD level (**a**) 0, (**b**) 1, (**c**) 2, and (**d**) 3.

We estimated the infection probability $b_{i}$ by fitting the actual confirmed cases for each age group by using a MATLAB-embedded function, *lsqcurvefit*, which is a nonlinear solver that finds the coefficient $b_{i}$ that minimizes gap of confirmed cases between the actual data and the simulated results from Equation (1) in the least-squares sense. The detailed explanation of the function is given in Supplementary Section B3. In the estimation, we used the contact matrix for each age group in Korea [36,43]. For SD level 0, 1, 2, and 3, to reflect the impacts of SD levels, we used different contact matrices $M_{0}$, $M_{1}$, $M_{2}$, and $M_{3}$, respectively. As shown by the parameter values in Table 4, the average time from the pre-symptomatic stage to confirmation is approximately 6 days. Thus, under the assumption that SD affects pre-symptomatic infection immediately, it is estimated that the number of confirmed cases is influenced approximately 6 days after the implementation of new SD. The details on the SD affecting confirmed cases and the infection probability $b_{i}$ are shown in Supplementary Section B4. Figure 4 and Table 5 show the results of the estimation of infection probability from the actual data of confirmed cases in the four distinct periods of SD levels 0, 1, 2, and 3. As SD level increases, the effective reproduction number $R_{t}$ decreases as shown in Table 5. The results of the confirmed cases fitting for all ages for each SD level are shown in Figure S2 in Supplementary Section C. The results of the (cumulative) confirmed cases fitting for each age group in different SD levels are shown in (Figures S7–S10) Figures S3–S6 in Supplementary Section C.

### 2.5. Ethical Considerations and Data Sharing Policy

COVID-19 data for Seoul and Gyeonggi Province are accessible in Reference [32,33], respectively. We used the fully anonymized data available in Reference [32,33], so there is no ethical issue to consider in this work.

## 3. Results

In this section, we investigated the effect of the age-dependent vaccination priority strategies under different levels of SD as described in Section 2.3. We assumed that 70% of the total population was vaccinated over 6 months, and the number of vaccinations per day was equal. 

**Figure 5:**
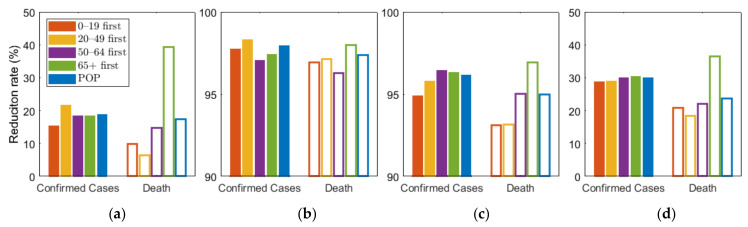
Effect of vaccination priority strategies for different SD levels (**a**) 0, (**b**) 1, (**c**) 2, (**d**) 3. Labels “0–19”, “20–49”, “50–64”, “65+” denote the vaccination priority strategies “0–19 first”, “20–49 first”, “50–64 first”, and “65+ first”, respectively.

As shown in Figure 5, we compared the effects of the vaccination priority strategies on the number of confirmed cases and deaths under different levels of SD. The exact values of the results displayed in Figure 5 are shown in Table 6.

Figure 5 shows that any vaccination strategies significantly reduced the number of incidences and mortalities, compared with no vaccination. Furthermore, Table 6 showed that any vaccination strategies under any SD level can reduce the number of incidences and fatalities by more than 10 times, compared with no SD at all. In particular, the highest SD level reduced the number by more than 4 times, compared with other SD levels. However, if the highest SD level persists for months, economic and social damage must be considered. Under SD level 0 or 1, when SD was minimally or not at all implemented, vaccination priority for the 20–49 age group reduced the cumulative incidence the most, and prioritizing the 65 or older group had the greatest reduction in the number of deaths. This result is consistent with the results of previous studies that did not assume SD [26]. However, under SD level 2 and 3, vaccination priority for 50–64 and 65 or older age groups reduced the cumulative confirmed cases the most, respectively. 

**Figure 6:**
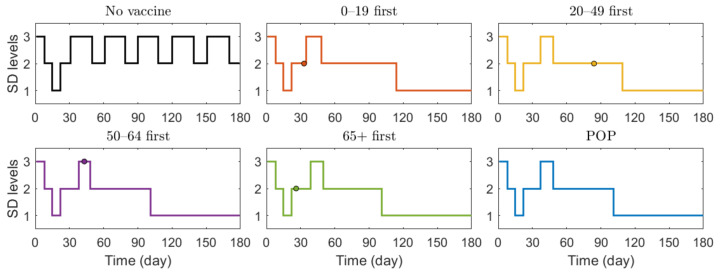
Transition of SD level according to the incidence under different scenarios.

**Figure 7:**
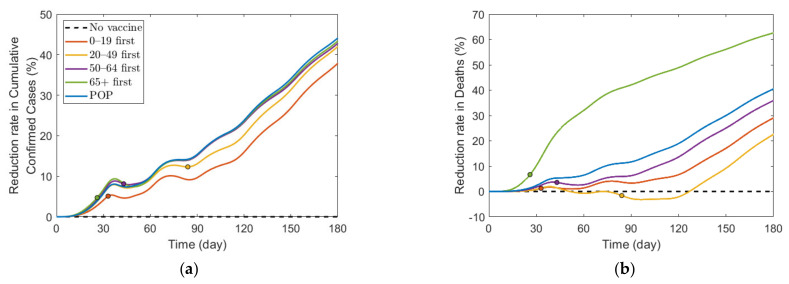
Effects of vaccination priority strategies when SD level changes adaptively according to the incidence. Time series of (**a**) reduction in cumulative confirmed cases and (**b**) reduction in deaths.

We also investigated the effect of vaccination priority strategies on the number of confirmed cases and deaths when the SD level changed adaptively according to the Korean governmental strategies [40]. The SD level changing criteria are presented in Table 3. Figure 6 illustrates the transition of SD level according to the incidence, and Figure 7 shows the reduction rates in confirmed cases and deaths for each vaccination strategies, compared with the case of no vaccination. The exact values of cumulative confirmed cases and deaths of each scenario in Figure 7 are shown in Table 7. The changes of SD level according to confirmed cases and SD level criteria when no vaccination was given is presented in Figure S11 in Supplementary Section C. Both in Figure 6 and Figure 7, the colored circles for strategies “0–19 first”, “20–49 first”, “50–64 first”, and “65+ first” denote the point when vaccination of the priority target population has reached 70%. Beyond this colored circle, vaccination was administered to other age groups proportional to their population until 70% of the total population was vaccinated.

Figure 6 shows that SD level was higher in the case of no vaccination than in other vaccination cases for most of the time, because, as shown in Figure 7, the number of confirmed cases in the case of no vaccination was much larger than that in other cases, making the SD level higher than that in other cases for most of the time. Figure 6 shows that without vaccination, SD level alternates between 2 and 3, while any vaccination strategy will lower the required SD level to 1. Figure 7 shows that reduction in death is substantially effective with strategy “65+ first”, compared with other strategies since vaccination targets the age group with the highest death rate, and any vaccination strategy successfully reduces the cumulative confirmed cases, compared with no vaccination scenario.

**Figure 8:**
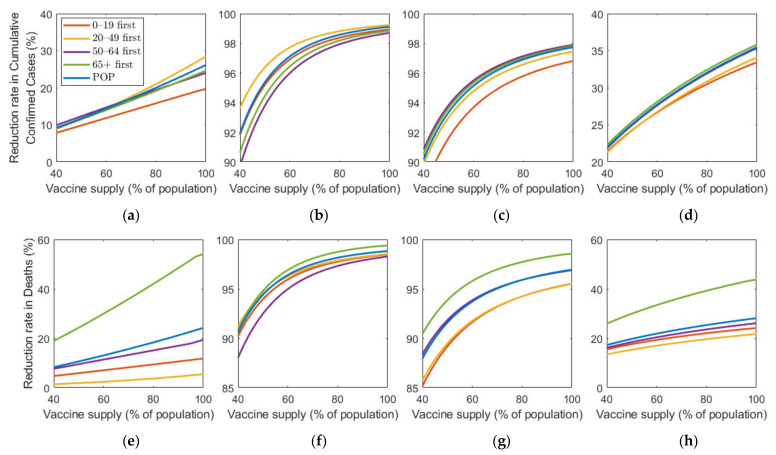
Impact of vaccination coverage rate on the reduction in cumulative confirmed cases and deaths for different SD levels: (**a**,**e**) 0, (**b**,**f**) 1, (**c**,**g**) 2, and (**d**,**h**) 3.

Figure 8 shows the effect of vaccine supply on the reduction in cumulative confirmed cases and mortalities for different SD levels. Instead of focusing on the vaccine strategy introduced previously at 70% coverage of the total population, we explored the effects for different vaccination coverages. As vaccine supply varied, we assumed that, in each vaccination priority strategy, vaccine was initially administered to the target age groups by x% of the target population and then administered to the other age groups proportionally to their population until x% of the total population was vaccinated, where x% ranged from 0% to 100%. Similarly, the POP strategy also considered variation in vaccine supply using the same total coverage as that in the other strategies.

Figure 8 shows that the reduction rate for the number of confirmed cases and deaths increased as vaccine supply rate increased in any SD levels. In particular, in the absence of SD, the reduction rate for the number of confirmed cases and deaths increased rapidly as the rate of vaccine supply increased. However, under SD, the reduction rate gradually increased with increasing vaccine supply. For the reduction in deaths, “65+ first” strategy was the most effective for any vaccine supply in all SD levels. However, the most effective strategy for reducing cumulative confirmed cases varied depending on SD level or supply. In SD level 0, when vaccine supply was relatively limited, “50–64 first” was the most effective strategy in reducing cumulative confirmed cases, but as supply increases, “20–49 first” was the best strategy. Strategies “20–49 first”, “50–64 first”, and “65+ first” were the most effective for any vaccine supply in SD level 1, 2, and 3, respectively, although the difference of reduction rate is minimal, compared with other strategies.

**Figure 9:**
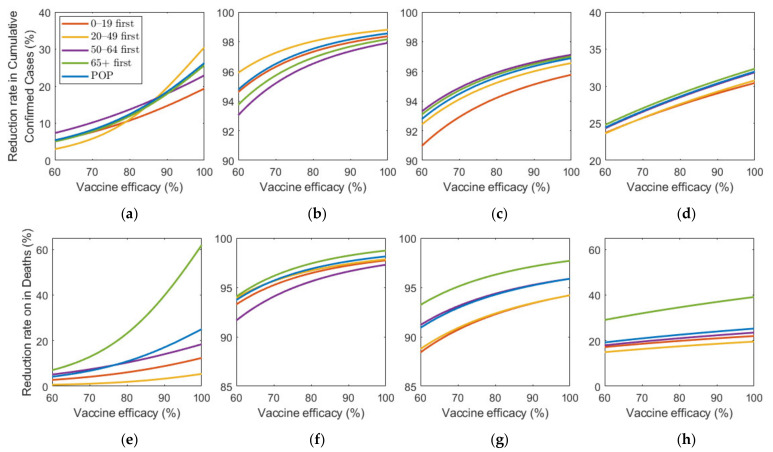
Impact of vaccination efficacy on the reduction in cumulative confirmed cases and deaths for different SD levels: (**a**,**e**) 0, (**b**,**f**) 1, (**c**,**g**) 2, and (**d**,**h**) 3.

Figure 9 shows the effect of vaccine efficacy on the reduction in cumulative confirmed cases and mortalities.

Figure 9 illustrates that an increase in vaccine efficacy resulted in an increase in reduction rate under any SD level. Similar to the results about vaccine supply, as the vaccine efficiency increased, the reduction rate of the number of confirmed cases and deaths increased much more rapidly in the absence of SD than in the case of SD. Moreover, “65+ first” strategy was the most effective for any vaccine efficacy in all SD levels for reducing deaths. The most effective strategy for reducing cumulative confirmed cases varied depending on SD level or vaccine efficacy. At SD level 0, when vaccine efficacy was relatively less effective, “50–64 first” was the most effective strategy in reducing cumulative confirmed cases, but with higher efficacy, “20–49 first” became the best strategy. Strategies “20–49 first”,”50–64 first”, and “65+ first” were the most effective for any vaccine supply in SD level 1, 2, and 3, respectively, although the reduction rate difference is minimal, compared with other strategies.

**Figure 10:**
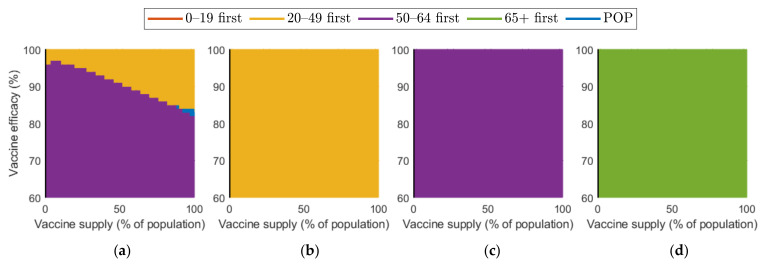
Vaccination priority strategy for the best reduction in cumulative confirmed cases of various vaccination supplies and efficacies under different SD levels: (**a**) 0, (**b**) 1, (**c**) 2, and (**d**) 3.

**Figure 11:**
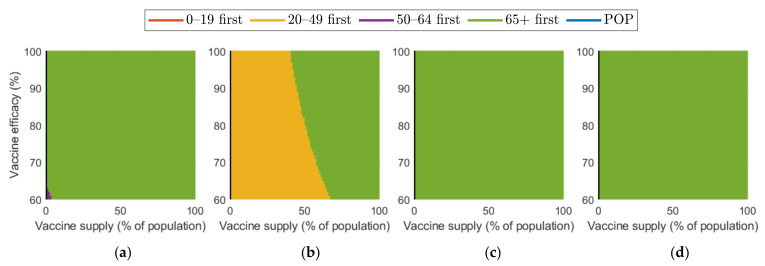
Effect of vaccination supply and efficacy on the reduction in mortality for different SD levels: (**a**) 0, (**b**) 1, (**c**) 2, and (**d**) 3.

Figure 10 and Figure 11 show the combined effect of vaccine supply and efficacy on the reduction in cumulative confirmed cases and mortalities, respectively. Given the vaccine supply and efficacy, the best vaccination priority strategy for reducing cumulative confirmed cases and deaths was found for each SD level.

Figure 10 shows that, in the case of no SD, “20–49 first” was the best strategy in terms of reducing the number of confirmed cases when both vaccine supply and efficacy were sufficiently large, but “50–64 first” gave the best reduction when vaccine supply and efficacy were relatively small. When SD level 1 was implemented, “20–49 first” resulted in the best reduction. However, in the case of SD level 2 and 3, “50–64 first” and “over 65 first” were the best strategy, respectively.

Regarding mortalities, Figure 11 illustrates that “over 65 first” was the best strategy for reducing the number of mortalities under any levels of SD, except for the case that vaccine supply was relatively low in SD levels 0 or 1.

**Figure 12:**
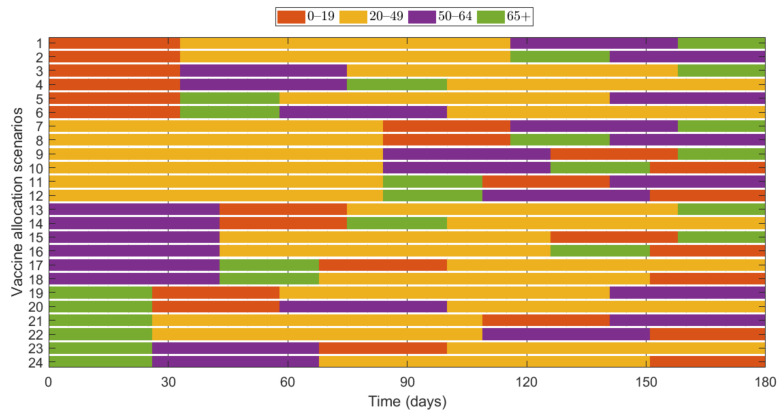
Scenarios of vaccination allocation orders.

**Figure 13:**
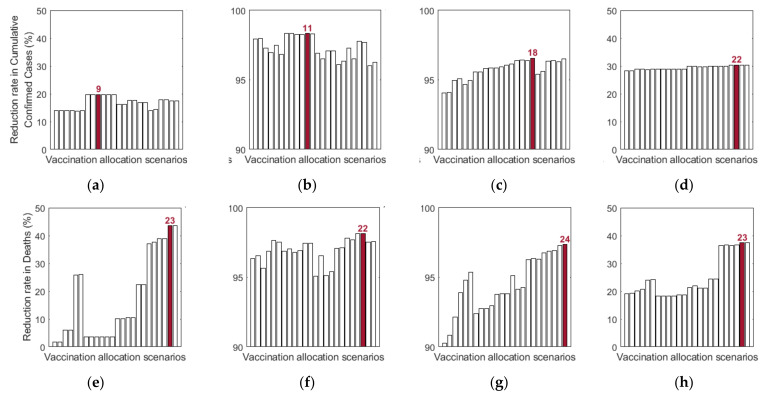
Impact of vaccination allocation scenarios on the reduction in cumulative confirmed cases and deaths for different SD levels: (**a**,**e**) 0, (**b**,**f**) 1, (**c**,**d**) 2, and (**d**,**h**) 3.

Additionally, we investigated the effects of vaccine allocation order based on the age groups 0–19, 20–49, 50–64, and 65+. Unlike the previous strategies, in which vaccine was distributed to the rest of the population after covering the target coverage rate of the initial age group, the next age group was selected for vaccination after the first group, where all four groups were vaccinated eventually. We assumed that only one group was vaccinated at a time with a coverage of 70% until the next vaccination group started vaccinating. Since there are four groups, there were 4!=24 different scenarios to vaccinate all the age groups in order. Like previous results, the effects of vaccine allocation order were examined based on reduction of the number of cumulative confirmed cases and deaths, compared with when vaccine was not given for each SD level. Figure 12 shows the details and orders of the vaccine administration for each scenario. Figure 13 shows the effects of each scenario at different SD levels. The optimal vaccine allocation order was determined for reducing cumulative confirmed cases and deaths for each SD level; the optimal results were colored red in Figure 13.

In Figure 13, for reducing the number of deaths, Scenario 23 (65+ → 50–64 → 0–19 → 20–49), Scenario 22 (65+ → 20–49 → 50–64 → 0–19), Scenario 24 (65+ → 50–64 → 20–49 → 0–19), and Scenario 23 (65+ → 50–64 → 0–19 → 20–49) were the most effective orders at SD level 0, 1, 2, and 3, respectively. That is, for any SD level, starting with the vaccination of the eldest age group 65+ yielded the best result for reducing mortalities. For reducing the number of cumulative confirmed cases, Scenario 9 (20–49 → 50–64 → 0–19 → 65+), Scenario 11 (20–49 → 65+ → 0–19 → 50–64), Scenario 18 (50–64 → 65+ → 20–49 → 0–19), and Scenario 22 (65+ → 20–49 → 50–64 → 0–19) were the most effective orders at SD level 0, 1, 2, and 3, respectively.

## 4. Discussion

In this study, we developed an age-structured mathematical model for describing the transmission of COVID-19, including vaccination. Using the model, we investigated the effect of vaccination priority strategies for different age groups on the transmission dynamics of COVID-19 under various scenarios of SD in Seoul/Gyeonggi area in Korea.

We estimated the infection probability of each age group under different levels of SD in the focus area by fitting the actual data of the confirmed cases by the least squares method. SD is one of the main NPIs implemented in Korea, and it played an important role in preventing a massive increase in the number of incidences and mortalities [36,37,38,39]. The effect of SD was reflected in terms of the contact matrix in our model, and the change in the number of confirmed patients due to direct/indirect effects of SD was used in the estimation of the infection probability. The change in the intensity of SD influenced the magnitude of the infection force, which was proportional to the infection probability and the entries of the contact matrix in the model Equation (1).

We observed that the reproduction number $R_{t}$ value decreased as the intensity of SD increased (Table 5), which shows the effect of NPIs implemented by the government in the course of our study.

To investigate the effect of vaccination in the target area, we reflected the vaccination plan prepared by the Korean government. The vaccination efficacy was calculated as 88%, based on the actual vaccine supply plan [8] in Korea and the efficacy of each vaccine as shown in Table 1. In the same manner as in the Korean government’s vaccination plan, we divided the total population into four age groups, 0–19, 20–49, 50–64, and 65 or older, and investigated the effect of various vaccination priorities for the age groups on the reduction in the number of confirmed cases and mortalities under different levels of SD implemented in Korea. We found that prioritization of COVID-19 vaccination for the eldest age group of 65 or older resulted in the greatest reduction in the total mortalities under any levels of SD. This result is consistent with WHO recommendations to prioritize COVID-19 vaccination for the elder age groups [49]. Recent studies suggested prioritizing vaccine allocation for younger age groups with higher contact rate to effectively reduce the incidence of infection [26], which is consistent with our results for the case that no or a low-level SD was implemented during the vaccination period. However, when a higher level of SD was implemented, our results showed that prioritizing COVID-19 vaccination for the elder age groups was the better strategy to reduce the number of confirmed cases. These results can be explained by the characteristics of the elderly life in Korea: when the intensity of SD is high, the elderly people who have close contacts in confined spaces, such as nursing homes and religious facilities, are more vulnerable to infection.

The Korean government has criteria for changing the intensity of SD according to the size of the number of confirm case [40]. By reflecting these criteria in the simulation, we investigated the effect of age-dependent vaccination priority strategies. The simulation results showed that vaccine priority for 65 or older group minimized the number of mortalities (Figure 7). One interesting thing is that, in the case of “20–49 first”, the reduction rate in mortality was lower than even that in the case of no vaccination. This can be explained by the fact that it takes more time to complete the vaccination at 70% for the “20–49” age group than for the other age group, as the former had the highest population ratio among all age groups [48], and thus vaccination for the elder age group with the highest mortality rate was delayed.

We investigated the effect of various vaccine supply rates on the reduction in the number of confirmed cases and mortalities (Figure 8). The results showed that the number of confirmed cases and deaths decreased very rapidly with increasing vaccine supply in the case of no SD, but, as the level of SD increased, the reduction rate in the number of confirmed cases and deaths decreased when vaccine supply increased. In other words, as the SD intensity decreased, the effectiveness of the vaccine supply in reducing the number of confirmed patients and deaths increased. Similar results were obtained qualitatively in the simulation about vaccine efficacy (Figure 9). Thus, in the case of no or low-level SD, a sufficient supply of vaccines with high efficacy is very important in reducing the number of confirmed cases and deaths.

We investigated the effect on the reduction in cumulative confirmed cases and deaths under different SD levels when vaccine supply and efficacy were considered together. Considering the currently expected vaccine supply and vaccine efficiency in Korea, “over 65 first” was the best strategy for reducing mortalities under all SD levels. Concerning the cumulative confirmed cases, “50–64 first” and “over 65 first” were the best strategy under SD level 2 and 3, respectively, but in SD level 1, prioritizing age group 20–49 gave the best result. In SD level 0, “50–64 first” was the best strategy in reducing the cumulative confirmed cases, except for the case that both of vaccine efficacy and supply are sufficiently large, for which “20–49 first” gave the best result.

Lastly, we examined the effects of vaccine allocation order on age groups 0–19, 20–49, 50–64, and 65+, where all four groups were vaccinated in succession with 70% coverage. The reduction in cumulative confirmed cases and deaths were tested for 24 different scenarios in each SD level. Depending on SD level, the optimal results varied, but for all SD levels, the optimal vaccination order for reducing deaths started with vaccinating the eldest group 65+.

The present study had some limitations. Priority vaccination for essential workers has been planned by the Korean government, but it is not considered in our simulation. Currently, the contact matrix is not available for essential workers related to COVID-19 in Korea; however, the total number of essential workers is only approximately 1% of the population in Korea. As we focused on the effect of vaccination priority strategy on different age groups, essential workers were not included in our model. Moreover, the infection probability in our model is affected by the contact matrix and incidence data, which differ by countries and regions. Thus, if the approach used in our study is applied to other countries or regions, the results would be different from ours.

Nevertheless, despite these limitations, we successfully analyzed the effect of the vaccination priority policy for different age groups on the reduction in the number of confirmed cases and mortalities by using our newly developed mathematical model, which reflected the actual SD policy and vaccination plan implemented in Korea. In addition, we investigated the effective age-dependent vaccination priority strategies to minimize the number of confirmed cases and deaths for various vaccine supplies and vaccine efficiencies. We believe that the modeling approach in this study can be used to investigate the potential effect of age-dependent vaccination priority strategies with various vaccine supplies and efficacies on the reduction in confirmed cases and mortalities in other target areas.

## Figures and Tables

**Table 1:** Description of vaccines.

Producer	Method	Efficacy	Total Number of Doses	Reference
Moderna “mRNA-1273”	mRNA base 2 doses, 4 weeks apart	94.1% 2 weeks after 2nd dose	40 million	[10]
Pfizer-BioNTech “BNT162b2”	mRNA base 2 doses, 3 weeks apart	95.0% 1 week after 2nd dose	26 million	[11]
Oxford University-AstraZeneca “AZD1222”	Viral vector base 2 doses, 4 weeks apart	62.1–90.0% 2 weeks after 2nd dose	20 million	[12]
Johnson & Johnson “Ad26.COV2.S”	Viral vector base 1 dose	57.0~72.0% (Overall 66.0%) 4weeks after dose	6 million	[13]
Novavax “NVX-CoV2373”	Protein-based 2 doses, 3 weeks apart	89.3% 1 week after 2nd dose	40 million	[14]

**Table 2:** Confirmed cases in Seoul/Gyeonggi area.

Age Group	Total	Region	
		Seoul	Gyeonggi
All age groups	47,371 (100.0%)	25,853 (54.6%)	21,518 (45.4%)
0–9	1987 (4.2%)	850 (3.3%)	1137 (5.3%)
10–19	2921 (6.2%)	1342 (5.2%)	1579 (7.3%)
20–29	6314 (13.3%)	3291 (12.7%)	3023 (14.0%)
30–39	6240 (13.2%)	3483 (13.5%)	2757 (12.8%)
40–49	6817 (14.4%)	3625 (14.0%)	3192 (14.8%)
50–59	8861 (18.7%)	4947 (19.1%)	3914 (18.2%)
60–69	7962 (16.8%)	4714 (18.2%)	3248 (15.1%)
70+	6269 (13.2%)	3601 (13.9%)	2668 (12.4%)

**Table 3:** Description of the modified social distancing (SD) in Korea.

SD Levels	Description	Criteria	Contact Matrix Variations
0	No SD	No criterion	No change
1	Corresponds to governmental SD level 1 and 1.5	Weekly average is less than 170 cases per day	Contact in locations other than workplace, household, and school decreased by 30%. Contact in household increased by 50% for age less than 20 and 10% for age 20 and above [44].
2	Corresponds to governmental SD level 2 and 2.5	At least 170 cases for 7 days	Contact in locations other than workplace, household, and school decreased by 50%. Contact in household increased by 50% for age less than 20 and 10% for age 20 and above [44].
3	Corresponds to governmental SD level 2.5 with reinforcements *	Weekly average is 280 or more cases per day	Contact in locations other than workplace, household, and school decreased by 70%. Contact in household increased by 50% for age less than 20 and 10% for age 20 and above [44].

* Governmental SD level 2.5 with reinforcements is assumed to be equivalent to governmental SD level 3 despite having slightly weaker standards [40] (Table S1 in Supplementary Section B1) due to stronger prohibition of gathering [40,42].

**Table 4:** Descriptions of parameters.

Parameter	Description	Value	References
$b_{i}$	Infection probability of a person in age group i per contact	Table 5	Estimated
$m_{ij}$	Number of contacts made by a person in age group j with people in age group i	Figure S1	[36]
$\phi_{i}$	Vaccination allocation for age group i	vary	Estimated
v	Daily vaccination doses	88,283	[8,9]
VP	Vaccination period	180	[45]
VC	Total vaccine coverage	0.7	[45]
τ	Vaccine efficacy	0.88	[10,11,12,13,14,45]
$\theta_{P}$	Relative infectiousness of pre-symptomatic infectious	0.51	[27]
$\theta_{A}$	Relative infectiousness of asymptomatic infectious	0.51	[27]
$\theta_{I}$	Relative infectiousness of symptomatically infectious	1	[27]
$1/\alpha_{E}$	Latent period $\left(day\right)$	3	[46]
$1/\alpha_{P}$	Pre-symptomatic period $\left(day\right)$	3.2	[46]
ρ	Probability of having symptoms	0.84	[27]
1/q	Mean duration of case confirmation $\left(day\right)$	3	[36]
$1/\gamma^{A}$	Recovery period of asymptomatic cases $\left(day\right)$	3.5	[27]
$1/\gamma_{i}^{M}$	Recovery period of mild symptom cases for group $i\left(day\right)$	* 15.3, 14.9, 16.3, 15.9, 15.5, 15.8, 16.5, 18.2	[32]
$1/\gamma_{i}^{S}$	Recovery period of severe symptom cases for group $i\left(day\right)$	* 15.3, 14.9, 16.3, 15.9, 15.5, 15.8, 16.5, 18.2	[32]
$\kappa_{i}$	Probability of having severe symptoms	0.26	[47]
$\mu_{i}$	Death rate of individuals in $H^{S}$ in age group i	* 0, 0, 0, 0, 0.001, 0.002, 0.009, 0.0832	[32]

* For cells with an asterisk (*), values from left to right at the top (bottom) are for age groups: 0–9, 10–19, 20–29, and 30–39 (40–49, 50–59, 60–69, and 70+).

**Table 5:** Values of infection probability and reproduction number depending on age group.

SD Level	Time Interval	Contact Matrix	$b_{i}\;*$	$R_{t}$
0	1 February– 22 February	$M_{0}$	3.97 × 10^−5^, 5.57 × 10^−2^, 7.82 × 10^−2^, 7.00 × 10^−2^, 5.78 × 10^−2^, 3.06 × 10^−2^, 1.12 × 10^−1^, 3.45 × 10^−1^.	3.6606
1	12 October– 23 November	$M_{1}$	3.43 × 10^−2^, 2.74 × 10^−2^, 2.84 × 10^−2^, 2.03 × 10^−2^, 2.15 × 10^−2^, 2.76 × 10^−2^, 7.73 × 10^−2^, 1.31 × 10^−1^.	1.4219
2	24 November– 22 December	$M_{2}$	2.99 × 10^−2^, 2.01 × 10^−2^, 2.28 × 10^−2^, 1.93 × 10^−2^, 1.93 × 10^−2^, 3.66 × 10^−2^, 1.08 × 10^−1^, 1.68 × 10^−1^.	1.2785
3	23 December– 14 February	$M_{3}$	2.64 × 10^−2^, 2.04 × 10^−2^, 1.78 × 10^−2^, 1.38 × 10^−2^, 1.33 × 10^−2^, 2.44 × 10^−2^, 7.45 × 10^−2^, 1.20 × 10^−1^.	0.8467

* In each cell for the fitted $b_{i}$, the values from left to right at the top (bottom) are for the age groups of 0–9, 10–19, 20–29, and 30–39 (40–49, 50–59, 60–69, and 70+), respectively.

**Table 6:** Values of cumulative confirmed cases and deaths depending on SD level and vaccination priority strategy.

Scenarios	Cumulative Confirmed Cases				Death			
	SD 0	SD 1	SD 2	SD 3	SD 0	SD 1	SD 2	SD 3
No Vaccine	16,820,437	6,378,361	1,315,182	9423	371,430	114,929	30,611	321
0–19 first	14,236,200	145,172	67,228	6716	335,020	3523	2109	254
20–49 first	13,162,693	106,477	55,142	6696	347,875	3309	2098	262
50–64 first	13,732,446	187,425	46,725	6604	316,673	4296	1521	250
65+ first	13,734,070	165,249	48,442	6557	225,287	2323	940	204
POP	13,670,909	131,505	50,502	6593	306,758	3009	1539	245

**Table 7:** Values of cumulative confirmed cases and deaths with SD levels changing adaptively.

Scenarios	Cumulative Confirmed Cases	Death
No Vaccine	50,538	1409
0–19 first	32,028	1000
20–49 first	29,361	1092
50–64 first	29,106	903
65+ first	28,774	527
POP	28,249	839

## Data Availability

The daily number of confirmed cases associated with COVID-19 in Seoul and Gyeonggi Province was accessed from publicly available sources, available at https://www.seoul.go.kr/coronaV/coronaStatus.do (accessed on 15 February 2021) and http://www.gidcc.or.kr/ (accessed on 15 February 2021).

## Supplementary Materials

The following are available online at https://www.mdpi.com/article/10.3390/ijerph18084240/s1, Section A1: Social Distancing (SD) in Korea (Table S1: Description of social distancing determined by Korean government); Section A2: Overall Vaccine Efficacy Calculation; Section B1: Contact Matrix (Figure S1: Contact matrix for different SD levels; SD level (a) 0,$\;M_{0}$, (b) 1,$\;M_{1}$, (c) 2,$\;M_{2}$, and (d) 3,$\;M_{3}$); Section B2: The Effective Reproduction Number $R_{t}$; Section B3: lsqcurvefit; Section B4: Infection Probability $b_{i}$; Section C: additional result figures (Figure S2: Estimation of transmission rate: confirmed cases of each SD level; Figure S3: Estimation of transmission rate: confirmed cases of each age group for SD level 0; Figure S4: Estimation of transmission rate: confirmed cases of each age group for SD level 1; Figure S5: Estimation of transmission rate: confirmed cases of each age group for SD level 2; Figure S6: Estimation of transmission rate: confirmed cases of each age group for SD level 3; Figure S7: Estimation of transmission rate: cumulative confirmed cases of each age group for SD level 0; Figure S8: Estimation of transmission rate: cumulative confirmed cases of each age group for SD level 1; Figure S9: Estimation of transmission rate: cumulative confirmed cases of each age group for SD level 2; Figure S10: Estimation of transmission rate: cumulative confirmed cases of each age group for SD level 3); Figure S11. Effects of vaccination priority strategies when SD level changes adaptively according to confirmed cases. Time series of (a) cumulative confirmed cases and (b) deaths.
