# Supplementary material for: Vaccination Prioritization Strategies for COVID-19 in Korea: A Mathematical Modeling Approach
Source: Int J Environ Res Public Health. 2021 Apr 16;18(8):4240. doi: 10.3390/ijerph18084240 (PMC8073596; doi:10.3390/ijerph18084240)
Supplement: Supplementary file 1 [file ijerph-18-04240-s001.zip › ijerph-1151858 Supplementary(1).pdf]

International Journal of
Environmental Research and Public Health

MDPI

Supplementary Section A.

In this section, we describe the detailed information regarding Korea's situation.

1. Social Distancing (SD) in Korea.

Table S1. Description of SD determined by Korean government [40]

Outbreak Scale	Level of SD	Description
Individual	0	▪ No SD.
Community	1	▪ Allows daily and socio-economic activities under basic level of epidemic prevention regulations.<br>▪ Prevalence is manageable under the capacity of the healthcare system.<br>▪ Weekly average is less than 100 cases per day.<br>▪ Cases from age 60 or more is less than 40 cases per day
Regional	1.5	▪ As local spread initiates, high risk areas follow strict level of epidemic prevention regulations. Prevalence in certain areas lasts more than a week threatening capacity of the healthcare system.<br>▪ Weekly average is more than 100 cases per day.<br>▪ Cases from age 60 or more is more than 40 cases per day
	2	▪ As local spreads rapidly expand, stay-at-home is advised while social meetings and visiting dense facilities in the public is refrained.<br>▪ Cases exceeds twice the standard even after the governmental SD level 1.5 for a week.<br>▪ Businesses for age 18+, including bars/clubs/casinos, are closed mandatorily.<br>▪ For two or more areas, governmental SD level 1.5 is continued for more than a week.<br>▪ National cases exceed 300 for more than a week.
National	2.5	▪ As epidemics is nationwide, stay-at-home is highly advised while social meetings and visiting all facilities in the public is refrained.<br>▪ Businesses are closed additionally including singing room, indoor concert halls/stages, and fitness centers.<br>▪ 400–500 cases daily across the nation.
	3	▪ As epidemics is nationwide, stay-at-home is highly advised and any contact with other people must be minimized.<br>▪ Prohibits the gathering of 10 or more people<br>▪ Most businesses and venues are closed including PC rooms, concert halls/stages, arcades, public bathhouses, museums, and movie theaters.<br>▪ 800–1000 cases daily across the nation.

2. Overall Vaccine Efficacy Calculation

The overall vaccine efficacy was calculated based on the vaccine supply plan as shown in Table 1. The weighted average was calculated using doses, where all vaccines except "Ad26.COV2.S" by Johnson & Johnson were divided by two, since these vaccines require two doses per person. Since "AZD1222" by Oxford University-AstraZeneca reported a mixture of results regarding efficacy depending on dose types [12], we assumed the midpoint of 62.1~90.0% as the efficacy of this vaccine.

$$\frac{\left[\begin{array}{c}94.1\% \cdot \frac{40 \text{ million doses}}{2 \text{ doses per person}} + 95.0\% \cdot \frac{26 \text{ million doses}}{2 \text{ doses per person}} + \frac{1}{2}(62.1 + 90.0)\% \cdot \frac{20 \text{ million doses}}{2 \text{ doses per person}} \\ +66.0\% \cdot \frac{6 \text{ million doses}}{1 \text{ doses per person}} + 89.3\% \cdot \frac{40 \text{ million doses}}{2 \text{ doses per person}}\end{array}\right]}{\left(\frac{40 \text{ million doses}}{2 \text{ doses per person}} + \frac{26 \text{ million doses}}{2 \text{ doses per person}} + \frac{20 \text{ million doses}}{2 \text{ doses per person}} + \frac{6 \text{ million doses}}{1 \text{ doses per person}} + \frac{40 \text{ million doses}}{2 \text{ doses per person}}\right)} \approx 88\%$$

Supplementary Section B.

In this section, we give further explanation of details on the methods.

1. Contact Matrix.

The contact matrix of Seoul and Gyeonggi Province, $C_M = (m_{i\,j})$ is an 8×8 matrix, where each element m_{ij} denotes the mean number of contacts an individual in age group i makes with individuals in age group j per day, where i and j are 10-year age groups, such as 0–9, 10–19, 20–29, 30–39, 40–49, 50–59, 60–69, and 70 and above.

C_M is a contact matrix formed by modifying the contact matrix estimated in [36] to match the 10-year age group. From [36], the 16×16 contact matrix of Seoul and Gyeonggi Province, $\tilde{C}_M$ is a contact matrix at given SD level where each element $\tilde{m}_{\tilde{\imath}\tilde{\jmath}}$ is the mean number of contacts an individual in age group $\tilde{\imath}$ makes with individuals in age group $\tilde{\jmath}$ per day. Here, $\tilde{\imath}$ and $\tilde{\jmath}$ are 5-year age groups, such as 0–4, 5–9, 10–14, 15–19, 20–24, 25–29, 30–34, 35–39, 40–44, 45–49, 50–54, 55–59, 60–64, 65–69, 70–74, and 75 and above.

By the definition of $\tilde{m}_{\tilde{\imath}\tilde{\jmath}}$, the $\tilde{t}_{\tilde{\imath}\tilde{\jmath}}$, the total number of contacts between age groups $\tilde{\imath}$ and $\tilde{\jmath}$ is

$$\tilde{t}_{\tilde{\imath}\tilde{\jmath}} = \tilde{m}_{\tilde{\imath}\tilde{\jmath}} \cdot \tilde{p}_{\tilde{\imath}},$$

where $\tilde{p}_{\tilde{\imath}}$ is the population of age group $\tilde{\imath}$. Note that $\tilde{m}_{\tilde{\imath}\,\tilde{\jmath}} \neq \tilde{m}_{\tilde{\jmath}\tilde{\imath}}$. Theoretically, it should be clear that the total number of contacts between age groups $\tilde{\imath}$ and $\tilde{\jmath}$ and the total number of contacts between age groups $\tilde{\jmath}$ and $\tilde{\imath}$ must be the same (i.e. $\tilde{t}_{\tilde{\imath}\,\tilde{\jmath}} = \tilde{t}_{\tilde{\jmath}\tilde{\imath}}$). However, since the contact matrix $\tilde{C}_M$ was initially modified from an estimated result in [43] with unknown population ratio for each age group, we found that $\tilde{t}_{\tilde{\imath}\,\tilde{\jmath}} \neq \tilde{t}_{\tilde{\jmath}\tilde{\imath}}$ for $\tilde{\imath} \neq \tilde{\jmath}$. Hence, we consider the total number of contact between age groups $\tilde{\imath}$ and $\tilde{\jmath}$ to be the average of $\tilde{t}_{\tilde{\imath}\,\tilde{\jmath}}$ and $\tilde{t}_{\tilde{\jmath}\tilde{\imath}}$, which is $\frac{1}{2}(\tilde{t}_{\tilde{\imath}\,\tilde{\jmath}} + \tilde{t}_{\tilde{\jmath}\tilde{\imath}})$.

We convert the age groups to 10-year age groups as follows:

1) Diagonal entries of C_M (for $i \neq j$) are

$$m_{ii} = \frac{\tilde{t}_{2i-1\,2i-1} + \frac{1}{2}(\tilde{t}_{2i-1\,2i} + \tilde{t}_{2i\,2i-1}) + \tilde{t}_{2i\,2i}}{\tilde{p}_{2i-1} + \tilde{p}_{2i}}$$
$$= \frac{\tilde{m}_{2i-1\,2i-1} \cdot \tilde{p}_{2i-1} + (\tilde{m}_{2i-1\,2i} \cdot \tilde{p}_{2i-1} + \tilde{m}_{2i\,2i-1} \cdot \tilde{p}_{2i})/2 + \tilde{m}_{2i\,2i} \cdot \tilde{p}_{2i}}{\tilde{p}_{2i-1} + \tilde{p}_{2i}},$$

2) Non-diagonal entries of C_M (for $\tilde{\imath} \neq \tilde{\jmath}$) are

$$m_{ij} = \frac{\frac{1}{2}\Big((\tilde{t}_{2i-1\,2j-1} + \tilde{t}_{2j-1\,2i-1}) + (\tilde{t}_{2i-1\,2j} + \tilde{t}_{2j\,2i-1}) + (\tilde{t}_{2i\,2j-1} + \tilde{t}_{2j-1\,2i}) + (\tilde{t}_{2i\,2j} + \tilde{t}_{2j\,2i})\Big)}{\tilde{p}_{2i-1} + \tilde{p}_{2i}}$$
$$= \frac{\begin{pmatrix}(\tilde{m}_{2i-1\,2j-1} \cdot \tilde{p}_{2i-1} + \tilde{m}_{2j-1\,2i-1} \cdot \tilde{p}_{2j-1}) + (\tilde{m}_{2i-1\,2j} \cdot \tilde{p}_{2i-1} + \tilde{m}_{2j\,2i-1} \cdot \tilde{p}_{2j}) \\ +(\tilde{m}_{2i\,2j-1} \cdot \tilde{p}_{2i} + \tilde{m}_{2j-1\,2i} \cdot \tilde{p}_{2j-1}) + (\tilde{m}_{2i\,2j} \cdot \tilde{p}_{2i} + \tilde{m}_{2j\,2i} \cdot \tilde{p}_{2j})\end{pmatrix}}{2(\tilde{p}_{2i-1} + \tilde{p}_{2i})}$$

Note that $\tilde{C}_M$ is calculated for each SD level, by taking the linear combination of location-specific matrices for workplace–m_W, school–m_S, household–m_H, and other locations– m_O obtained from [43] using the modifications in [36]; a brief explanation of the modification is shown in Table 3.

$$\tilde{C}_M = c_W \cdot m_W + c_S \cdot m_S + c_H \cdot m_H + c_O \cdot m_O,$$

where c_W, c_S, and c_O are constants, and c_H is a 16×16 diagonal matrix with n diagonal entries. To summarize the modifications in [36], for all scenarios no modifications were made for the workplace ($c_W = 1$) and school was assumed to be closed ($c_S = 0$). When SD was implemented, contact in household increased ($c_H = diag(1.5, 1.5, 1.5, 1.5, 1.1, 1.1, \ldots, 1.1)_{16}$) identically for SD levels 1,2, and 3. The only difference between SD levels occur in other locations ($c_O = 0.3, 0.5, 0.7$ for SD levels 1, 2, and 3, respectively). Eventually, M_0, M_1, M_2, and M_3 are 8×8

matrices modified using the above method from the 16× 16 contact matrices calculated in [36], where each SD level corresponds in the following way: SD level 0 – School Closing/No Social Distancing, SD level 1 – School Closing/Weak Social Distancing, SD level 2 – School Closing/Medium Social Distancing, and SD level 3 – School Closing/Strong Social Distancing.

	0-9	10-19	20-29	30-39	40-49	50-59	60-69	70+
0-9	1.54	0.67	0.43	1.15	0.64	0.33	0.30	0.24
10-19	0.79	4.63	1.13	0.96	1.41	0.63	0.26	0.40
20-29	0.76	1.67	4.57	2.59	1.96	1.79	0.69	0.35
30-39	2.12	1.51	2.74	4.41	2.97	1.95	1.27	0.73
40-49	1.32	2.48	2.32	3.32	3.55	2.34	1.03	1.01
50-59	0.65	1.07	2.05	2.11	2.26	2.40	1.03	0.69
60-69	0.41	0.31	0.55	0.96	0.69	0.71	0.96	0.50
70+	0.27	0.38	0.22	0.44	0.55	0.39	0.40	0.55

(a)

	0-9	10-19	20-29	30-39	40-49	50-59	60-69	70+
0-9	1.77	0.75	0.47	1.40	0.77	0.35	0.36	0.31
10-19	0.88	4.56	1.03	1.01	1.67	0.74	0.30	0.52
20-29	0.67	1.40	4.12	2.28	1.80	1.66	0.61	0.30
30-39	2.03	1.36	2.41	4.06	2.64	1.71	1.12	0.63
40-49	1.26	2.38	2.13	2.96	3.34	2.09	0.86	0.89
50-59	0.58	1.02	1.90	1.85	2.02	2.27	0.85	0.60
60-69	0.39	0.29	0.49	0.84	0.58	0.59	0.91	0.40
70+	0.27	0.38	0.19	0.38	0.48	0.34	0.32	0.51

(b)

	0-9	10-19	20-29	30-39	40-49	50-59	60-69	70+
0-9	1.63	0.69	0.42	1.32	0.72	0.31	0.33	0.30
10-19	0.81	3.98	0.89	0.92	1.58	0.69	0.28	0.50
20-29	0.59	1.20	3.73	2.05	1.67	1.54	0.54	0.25
30-39	1.88	1.22	2.17	3.75	2.41	1.54	0.99	0.54
40-49	1.16	2.22	1.97	2.70	3.14	1.92	0.74	0.78
50-59	0.50	0.95	1.76	1.67	1.85	2.12	0.72	0.51
60-69	0.36	0.26	0.43	0.74	0.50	0.50	0.84	0.33
70+	0.26	0.36	0.16	0.33	0.42	0.29	0.27	0.47

(c)

	0-9	10-19	20-29	30-39	40-49	50-59	60-69	70+
0-9	1.50	0.62	0.37	1.24	0.67	0.28	0.31	0.29
10-19	0.73	3.41	0.76	0.84	1.49	0.65	0.26	0.48
20-29	0.51	0.99	3.34	1.83	1.53	1.42	0.48	0.21
30-39	1.73	1.09	1.94	3.43	2.17	1.37	0.86	0.45
40-49	1.06	2.06	1.82	2.43	2.93	1.74	0.62	0.66
50-59	0.43	0.88	1.62	1.48	1.68	1.97	0.59	0.43
60-69	0.33	0.24	0.38	0.64	0.42	0.41	0.77	0.26
70+	0.25	0.34	0.14	0.27	0.36	0.24	0.21	0.42

(d)

Figure S1. Contact matrix for different SD levels; SD level (a) 0, M_0, (b) 1, M_1, (c) 2, M_2, and (d) 3, M_3.

2. The Effective Reproduction Number R_t.

The system has the disease-free state $x_0 = (S_i, 0,0,0,0,0,0,0,0,0)$. Let $x = (E_i, P_i, A_i, I_i, H_i^M, H_i^S)^T$ for $i = 1,2,..,8$. $F(x)$ represents all of the new infections. The net transition rates of the corresponding compartments are represented by $V(x)$.

$$F(x) = \begin{pmatrix} (S_i + (1-\tau)V_i)\Lambda_i \\ 0 \\ 0 \\ 0 \\ 0 \\ 0 \end{pmatrix}$$

where $\Lambda_i = b_i \sum_j^8 \left[\frac{m_{ij}(\theta_P P_j + \theta_A A_j + \theta_I I_j)}{N_j}\right]$, $N_j = S_j + E_j + P_j + A_j + I_j + R_j$.

$$V(x) = \begin{pmatrix} \alpha_E E_i \\ -\alpha_E E_i + \alpha_P P_i \\ -(1-\rho)\alpha_P P_i + \gamma^A A_i \\ -\rho\alpha_P P_i + qI_i \\ -(1-\kappa_i)qI_i + \gamma_i^L H_i^M \\ -\kappa_i q I_i + \gamma_i^S H_i^S + \mu_i H_i^S \end{pmatrix}.$$

Thus, **F** and **V** are $48 * 48$ matrices at x_0 given by

$$\mathbf{F}(x) = \begin{bmatrix} 0_{8,8} & \theta_P M_A & \theta_A M_A & \theta_I M_A & 0_{8,8} & 0_{8,8} \\ 0_{8,8} & 0_{8,8} & 0_{8,8} & 0_{8,8} & 0_{8,8} & 0_{8,8} \\ 0_{8,8} & 0_{8,8} & 0_{8,8} & 0_{8,8} & 0_{8,8} & 0_{8,8} \\ 0_{8,8} & 0_{8,8} & 0_{8,8} & 0_{8,8} & 0_{8,8} & 0_{8,8} \\ 0_{8,8} & 0_{8,8} & 0_{8,8} & 0_{8,8} & 0_{8,8} & 0_{8,8} \\ 0_{8,8} & 0_{8,8} & 0_{8,8} & 0_{8,8} & 0_{8,8} & 0_{8,8} \end{bmatrix}$$

Here M_A is the matrix computed as

$$M_A = diag\{b_1(S_1 + (1-\tau)V_1), b_2(S_2 + (1-\tau)V_2), \ldots, b_8(S_8 + (1-\tau)V_8)\}_8 * C_M * diag\left\{\frac{1}{N_1}, \frac{1}{N_2}, \ldots, \frac{1}{N_8}\right\}_8,$$

where S_i is the susceptible population of age group i, V_i is the vaccinated population of age group i, C_M is the 8×8 contact matrix, and $diag\{\}_n$ denotes the diagonal matrix with n diagonal entries.

$$\mathbf{V}(x) = \begin{bmatrix} M_B & 0_{8,8} & 0_{8,8} & 0_{8,8} & 0_{8,8} & 0_{8,8} \\ -M_B & M_C & 0_{8,8} & 0_{8,8} & 0_{8,8} & 0_{8,8} \\ 0_{8,8} & -(1-\rho)M_C & M_D & 0_{8,8} & 0_{8,8} & 0_{8,8} \\ 0_{8,8} & -\rho M_C & 0_{8,8} & M_E & 0_{8,8} & 0_{8,8} \\ 0_{8,8} & 0_{8,8} & 0_{8,8} & -(1-\kappa_i)M_E & M_F & 0_{8,8} \\ 0_{8,8} & 0_{8,8} & 0_{8,8} & -\kappa_i M_E & 0_{8,8} & M_G \end{bmatrix}$$

where $M_B = \alpha_E * \mathbf{I}_8$, $M_C = \alpha_P * \mathbf{I}_8$, $M_D = \gamma^A * \mathbf{I}_8$, $M_E = q * \mathbf{I}_8$, $M_F = \gamma_i^L * \mathbf{I}_8$, $M_G = (\gamma_i^S + \mu_i) * \mathbf{I}_8$, and $\mathbf{I}_8$ is the size 8 identity matrix.

Then, the inverse matrix of **V** is

$$\mathbf{V}^{-1}(x) = \begin{bmatrix} M_B^{-1} & 0_{8,8} & 0_{8,8} & 0_{8,8} & 0_{8,8} & 0_{8,8} \\ M_C^{-1} & M_C^{-1} & 0_{8,8} & 0_{8,8} & 0_{8,8} & 0_{8,8} \\ (1-\rho)M_D^{-1} & (1-\rho)M_D^{-1} & M_D^{-1} & 0_{8,8} & 0_{8,8} & 0_{8,8} \\ \rho M_E^{-1} & \rho M_E^{-1} & 0_{8,8} & M_E^{-1} & 0_{8,8} & 0_{8,8} \\ \rho(1-\kappa_i)M_F^{-1} & \rho(1-\kappa_i)M_F^{-1} & 0_{8,8} & (1-\kappa_i)M_F^{-1} & M_F^{-1} & 0_{8,8} \\ \rho\kappa_i M_G^{-1} & \rho\kappa_i M_G^{-1} & 0_{8,8} & \kappa_i M_G^{-1} & 0_{8,8} & M_G^{-1} \end{bmatrix}.$$

Hence, one can obtain the next generation matrix $\boldsymbol{G}$ as

$$\boldsymbol{G} = \mathbf{FV}^{-1} = \begin{bmatrix} \left(\frac{\theta_P}{\alpha_P} + \frac{(1-\rho)\theta_A}{\gamma^A} + \frac{\rho\theta_I}{q}\right) M_A & \left(\frac{\theta_P}{\alpha_P} + \frac{(1-\rho)\theta_A}{\gamma^A} + \frac{\rho\theta_I}{q}\right) M_A & \frac{\theta_A}{\gamma^A} M_A & \frac{\theta_I}{q} M_A & 0_{8,8} & 0_{8,8} \\ 0_{8,8} & 0_{8,8} & 0_{8,8} & 0_{8,8} & 0_{8,8} & 0_{8,8} \\ 0_{8,8} & 0_{8,8} & 0_{8,8} & 0_{8,8} & 0_{8,8} & 0_{8,8} \\ 0_{8,8} & 0_{8,8} & 0_{8,8} & 0_{8,8} & 0_{8,8} & 0_{8,8} \\ 0_{8,8} & 0_{8,8} & 0_{8,8} & 0_{8,8} & 0_{8,8} & 0_{8,8} \\ 0_{8,8} & 0_{8,8} & 0_{8,8} & 0_{8,8} & 0_{8,8} & 0_{8,8} \end{bmatrix}.$$

Finally, the effective reproduction number R_t is computed as the spectral radius $\boldsymbol{\rho}(\boldsymbol{G})$ of the next generation matrix $\boldsymbol{G}$, $i.e. R_t = \boldsymbol{\rho}(\boldsymbol{G})$.

$$R_t = \boldsymbol{\rho}(\boldsymbol{G}) = \left(\frac{\theta_P}{\alpha_P} + \frac{(1-\rho)\theta_A}{\gamma^A} + \frac{\rho\theta_I}{q}\right) \boldsymbol{\rho}(\boldsymbol{M_A}).$$

3. lsqcurvefit

In order to find the best fitting value of b_i, we use *MATLAB*-embedded function, *lsqcurvefit*. In our case, the function is a nonlinear solver that finds the 8×1 coefficient vector $= (b_1, b_2, \ldots, b_8)$, which is the set of infection probabilities for age groups 0–9, 10–19, 20–29, 30–39, 40–49, 50–59, 60–69, and 70+, that solves the problem

$$\min_{\boldsymbol{b}} \|\mathbf{F}(\boldsymbol{b}) - \mathbf{C}\|_2^2 = \min_{\boldsymbol{b}} \sum_t \left(F(\boldsymbol{b}, t) - C(t)\right)^2 \qquad (*)$$

Here, $\mathbf{F}(\boldsymbol{b})$ gives the simulated number of confirmed cases for all age groups during the whole period using $\boldsymbol{b}$ as infection probability with $\dim\left(\mathbf{F}(\boldsymbol{b})\right) = 8 \times (\text{length of period})$. $\mathbf{C}$ is the actual number of confirmed cases during the whole period with $\dim(\mathbf{C}) = 8 \times (\text{length of period})$. $F(\boldsymbol{b}, t)$ gives the number of confirmed cases for all age groups at time *t* using $\boldsymbol{b}$ as infection probability, and $C(t)$ is the actual number of confirmed cases at time *t* with $\dim\left(\mathrm{F}(\boldsymbol{b}, t)\right) = \dim\left(C(t)\right) = 8 \times 1$.

In other words, we found the set of infection probabilities for different age groups that minimizes the error in the least-squares sense between real confirmed cases data when simulated. Namely, lsqcurvefit$(\mathbf{F}, \boldsymbol{b_0}, \boldsymbol{t}, \mathbf{C})$ returns $\boldsymbol{b}$ satisfying $(*)$, where $\boldsymbol{b_0}$ is the initial value of $\boldsymbol{b}$.

4. Infection Probability b_i.

The infection probability b_i from Table 5 denotes the infection probability of a person in age group i per contact, where i is a 10-year age group such as 0–9, 10–19, 20–29, 30–39, 40–49, 50–59, 60–69, and 70 or older (70+). Each individual in age group i will make contact with individuals in age group j based on the population in each compartment at given time and the contact matrix, where i and j are both 10-year age groups. If the contacting person in age group j is infectious (i.e. belongs to compartment $P_j, A_j,$ or I_j), then a susceptible person in age group i, S_i, will be infected based on this probability. Note that b_i determines the potential of an individual being infected given the contact with an infectious person [36,43]. So, we consider b_i as a product of susceptibility and behavior affecting infection probability. Susceptibility is a trait fixed to a person based on their age while behavior varies with time. Even with the same contact, depending on SD level, individuals may recognize the situation differently which can affect the behavior and eventually their probability of infection. More specifically, different SD levels can impact individual behaviors differently due to different guidelines.[1] Well-known guidelines encompassing wearing masks, making less conversation in public, keeping physical distance with others, frequent ventilation, washing hands, and coughing etiquettes can affect one's infection probability given the same contact. Thus, we interpret b_i as a parameter which connotes susceptibility and SD level based behavior, and b_i is estimated for each SD level periods for each 10-year age groups.

For a person to be diagnosed as confirmed with COVID-19, the person must have undergone two states: pre-symptomatically infectious and symptomatically infectious. As SD is intended to reduce the contact between people, a person who is newly diagnosed as confirmed is most likely to be infected by the policy of SD when the contact with an infection person happened. Since SD affects both the contact between the infector and the infectee, we must consider the pre-symptomatic period, $1/\alpha_P$, and the mean duration of the case confirmation, $1/q$. Thus, by the sum of the two periods based on the values in Table 4, we get 6.2 days. Since, confirmed cases are reported daily, we instead use 6 days for the delay.

[1] Kim, Eun-A. "Social Distancing and Public Health Guidelines at Workplaces in Korea: Responses to Coronavirus Disease-19." *Safety and Health at Work* 11.3 (2020): 275-283.

Supplementary Section C.

In this section, we show the result figures.

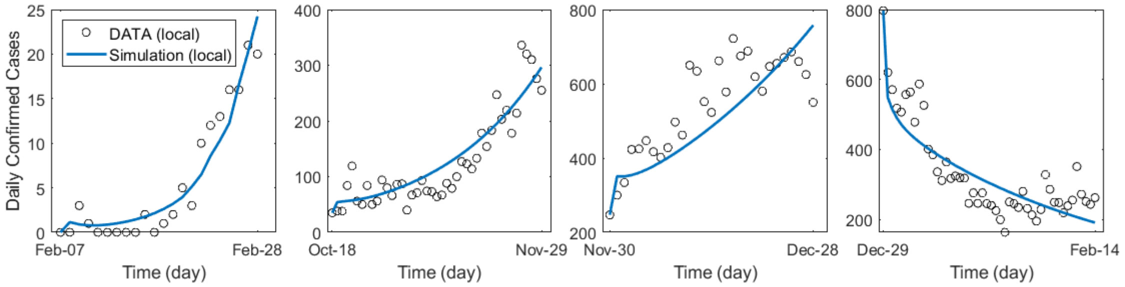

Figure S2. Estimation of infection probability: confirmed cases of each SD level.

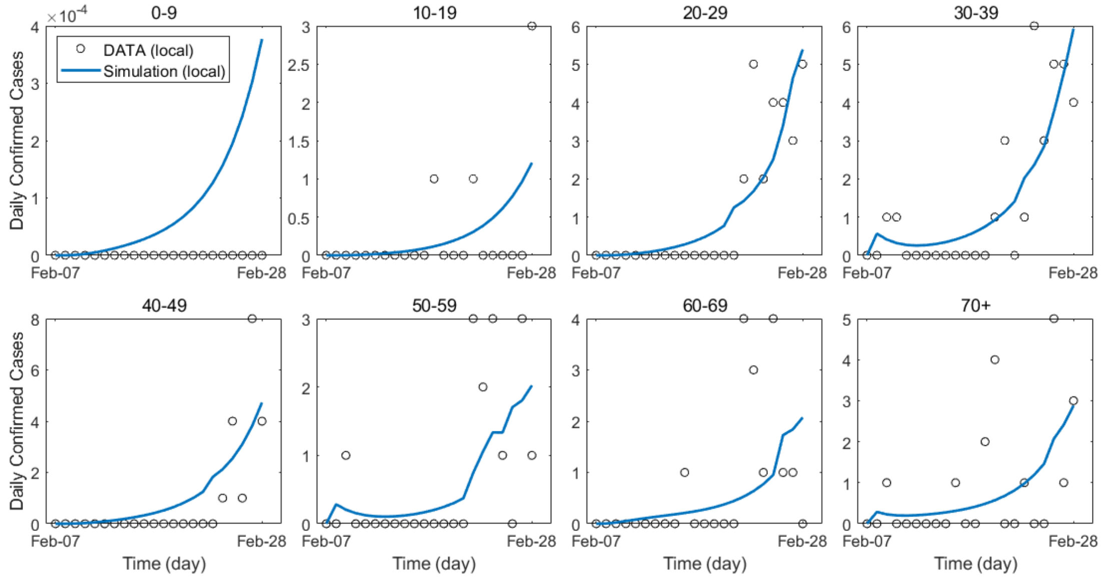

Figure S3. Estimation of infection probability: confirmed cases of each age group for SD level 0.

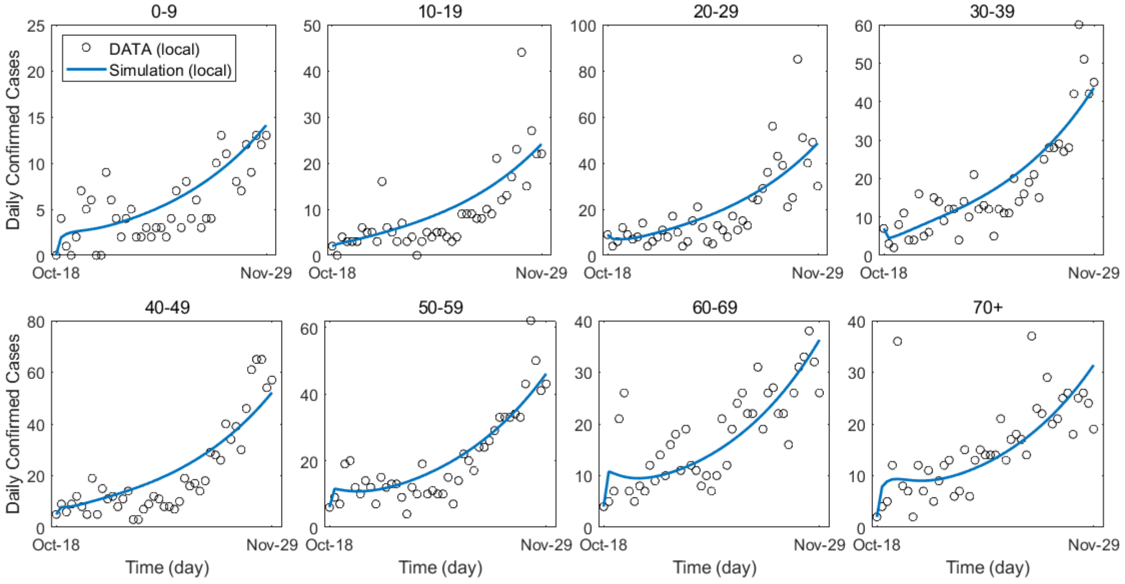

Figure S4. Estimation of infection probability: confirmed cases of each age group for SD level 1.

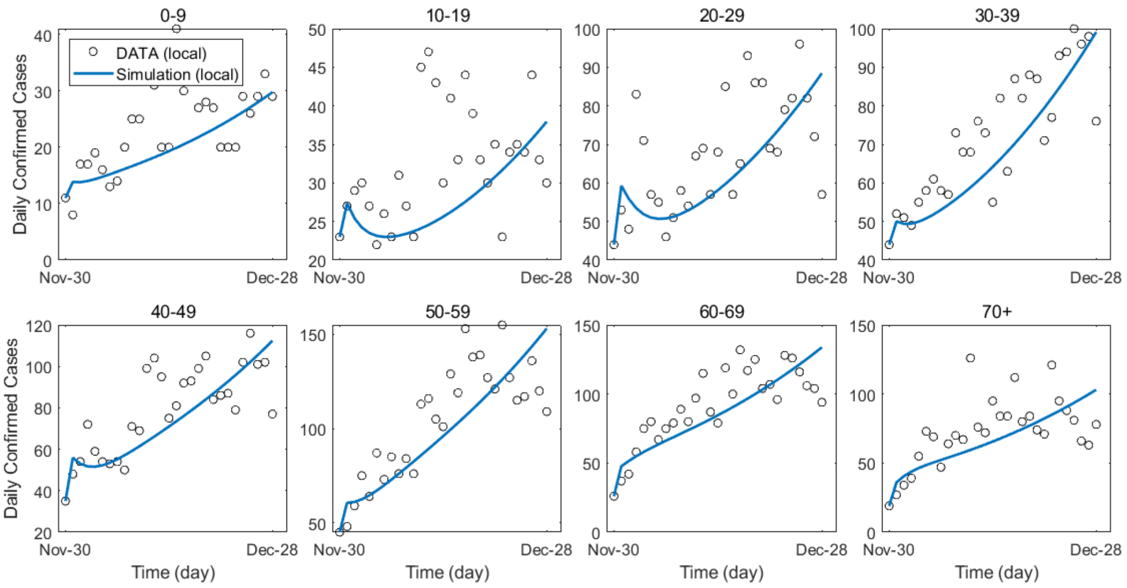

Figure S5. Estimation of infection probability: confirmed cases of each age group for SD level 2.

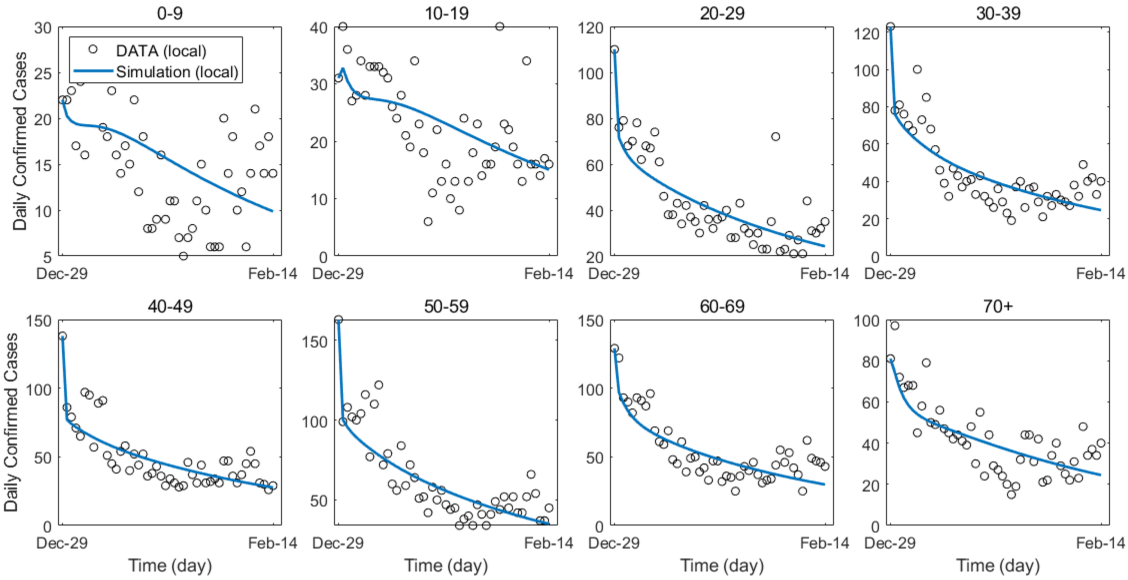

Figure S6. Estimation of infection probability: confirmed cases of each age group for SD level 3.

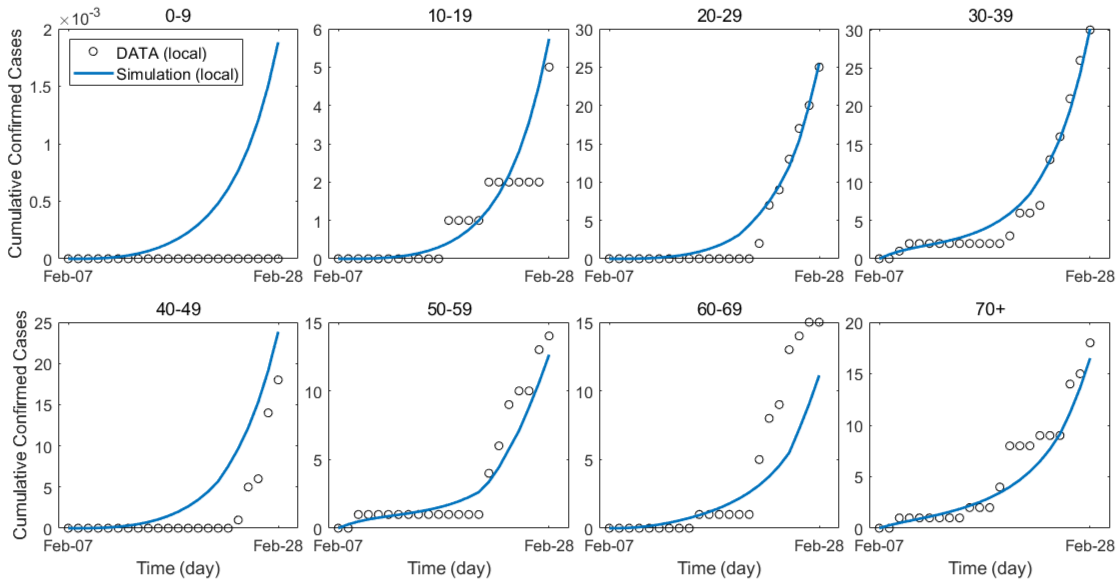

Figure S7. Estimation of infection probability: cumulative confirmed cases of each age group for SD level 0.

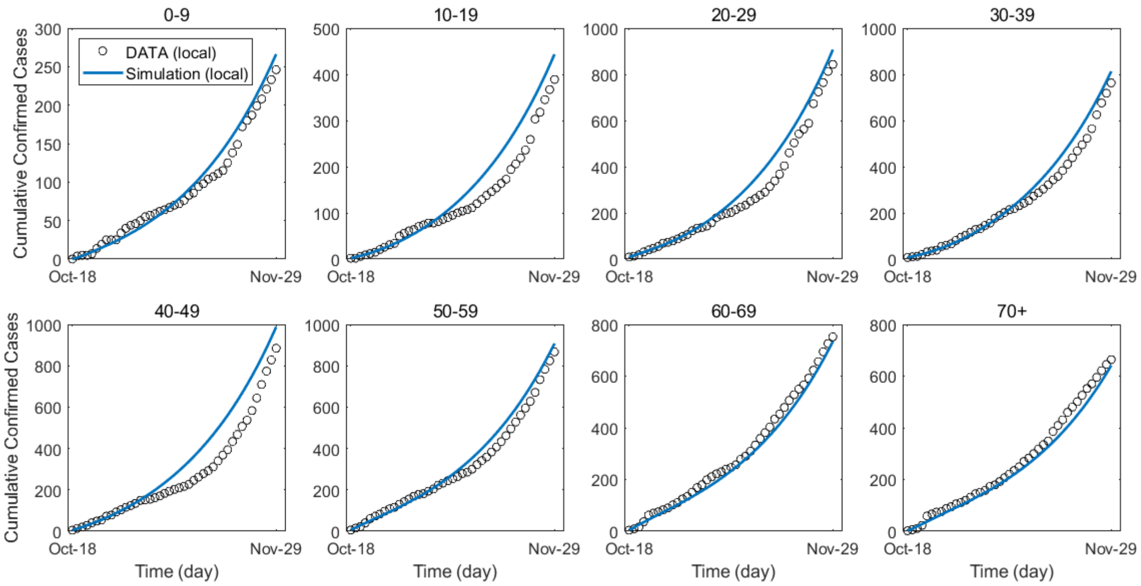

Figure S8. Estimation of infection probability: cumulative confirmed cases of each age group for SD level 1.

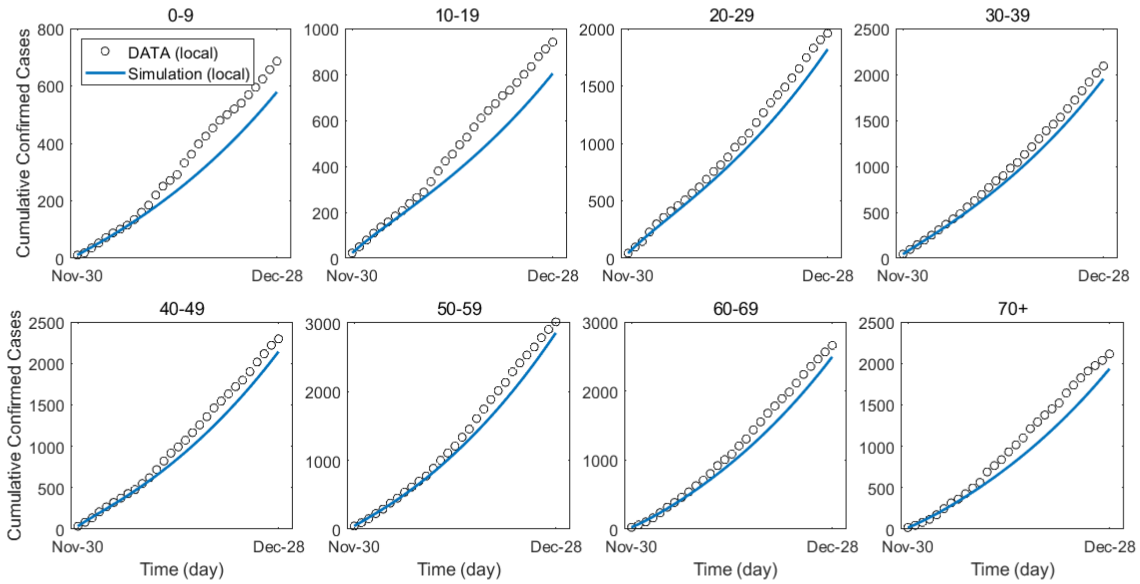

Figure S9. Estimation of infection probability: cumulative confirmed cases of each age group for SD level 2.

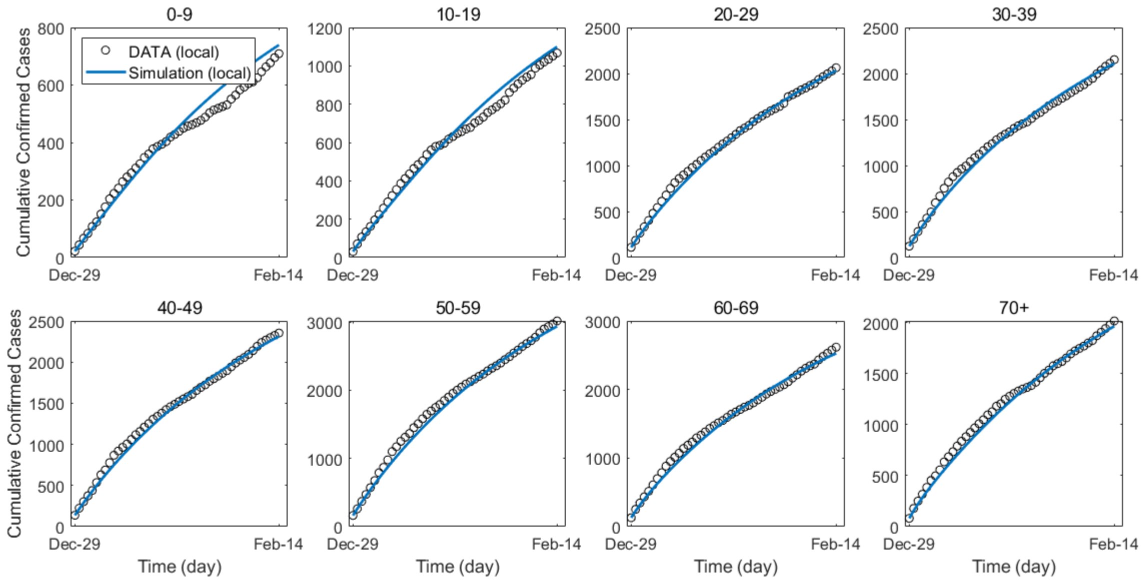

Figure S10. Estimation of infection probability: cumulative confirmed cases of each age group for SD level 3.

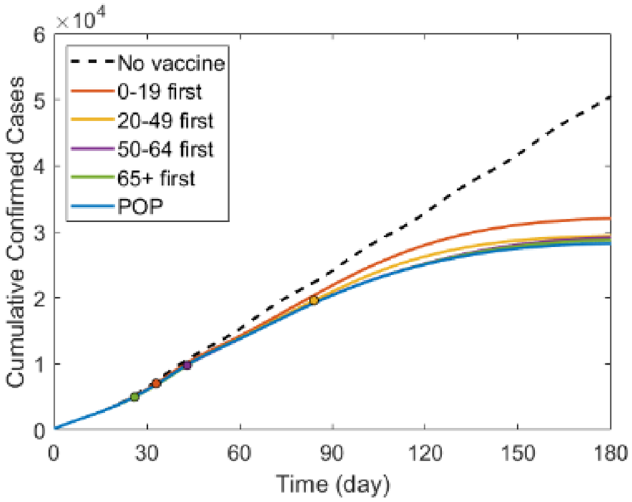

(a)

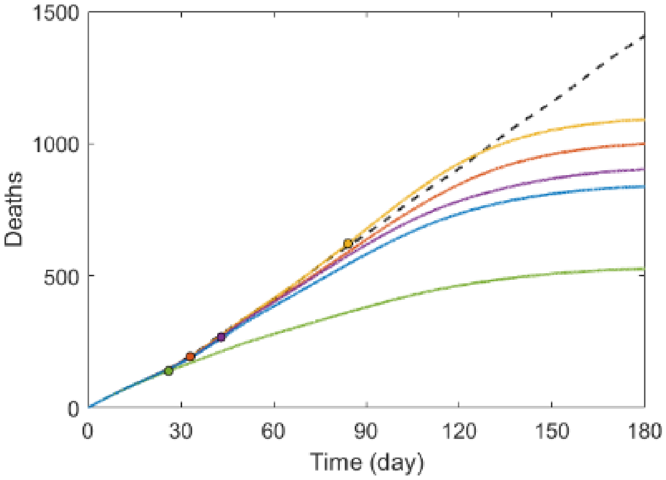

(b)

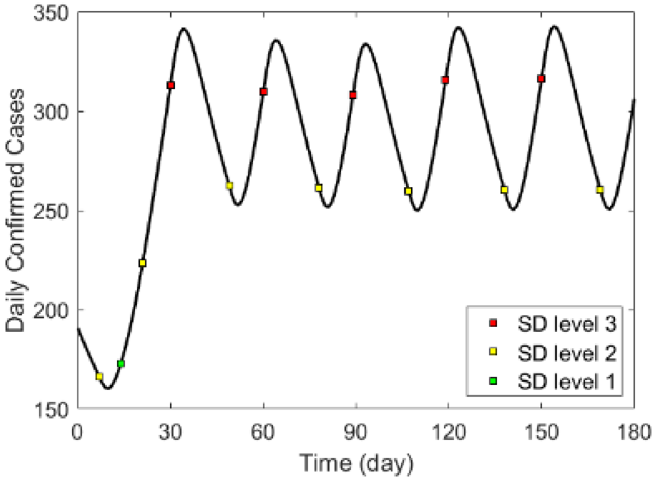

(c)

Figure S11. Effects of vaccination priority strategies when SD level changes adaptively according to confirmed cases. Time series of (a) cumulative confirmed cases and (b) deaths. (c) Time series of daily confirmed cases with no vaccination; note that SD level shifting occurs at colored points based on the criteria in Table 3.
